# The gain and loss of genes during 600 million years of vertebrate evolution

**DOI:** 10.1186/gb-2006-7-5-r43

**Published:** 2006-05-24

**Authors:** Tine Blomme, Klaas Vandepoele, Stefanie De Bodt, Cedric Simillion, Steven Maere, Yves Van de Peer

**Affiliations:** 1 Department of Plant Systems Biology, Flanders Interuniversity Institute for Biotechnology (VIB), Ghent University, Technologiepark, B-9052 Ghent, Belgium

## Abstract

**Background:**

Gene duplication is assumed to have played a crucial role in the evolution of vertebrate organisms. Apart from a continuous mode of duplication, two or three whole genome duplication events have been proposed during the evolution of vertebrates, one or two at the dawn of vertebrate evolution, and an additional one in the fish lineage, not shared with land vertebrates. Here, we have studied gene gain and loss in seven different vertebrate genomes, spanning an evolutionary period of about 600 million years.

**Results:**

We show that: first, the majority of duplicated genes in extant vertebrate genomes are ancient and were created at times that coincide with proposed whole genome duplication events; second, there exist significant differences in gene retention for different functional categories of genes between fishes and land vertebrates; third, there seems to be a considerable bias in gene retention of regulatory genes towards the mode of gene duplication (whole genome duplication events compared to smaller-scale events), which is in accordance with the so-called gene balance hypothesis; and fourth, that ancient duplicates that have survived for many hundreds of millions of years can still be lost.

**Conclusion:**

Based on phylogenetic analyses, we show that both the mode of duplication and the functional class the duplicated genes belong to have been of major importance for the evolution of the vertebrates. In particular, we provide evidence that massive gene duplication (probably as a consequence of entire genome duplications) at the dawn of vertebrate evolution might have been particularly important for the evolution of complex vertebrates.

## Abstract 2

Phylogenetic analysis of gene gain and loss during vertebrate evolution provides evidence for the importance of early gene or genome duplication events in evolution of complex vertebrates.

## Background

The sequencing of vertebrate genomes occurs at an ever-increasing pace. Currently, the genome sequences, or at least first drafts thereof, are available for more than 14 different vertebrate species, while many more are underway. These vertebrate genome sequences cover a phylogenetic distance of more than 450 million years of evolution, dating back as far as the split between fishes and land vertebrates. Unfortunately, genome sequences of cartilaginous fish such as sharks, rays or skates, or of jawless vertebrates such as lampreys and hagfish, which diverged well before that time, are not available yet.

Based on rather inaccurate indicators such as genome size and isozyme complexity, Ohno already suggested in 1970 that the genomes of (early) vertebrates have been shaped by two whole genome duplications (WGDs) [1]. More than 20 years later, important indications for two rounds (1R/2R) of large-scale gene duplications in early vertebrate evolution came from the analysis of *Hox *genes and *Hox *gene clusters [2-4]. Since then, the 2R hypothesis has been heavily debated, and several modifications have been proposed, assuming a diversity of small and large scale gene duplication events (reviewed in [5]). Based on quadruplicate paralogy between different genomic segments [6-8], or a large increase in the number of new duplicated genes at the dawn of vertebrate evolution about 600 million years ago (MYA) [9], some have indeed strongly argued for two rounds of genome duplications, possibly in very short succession [10,11]. Others, often analyzing the same data but using different techniques, found only clear evidence for one genome-doubling event [12-14]. Still others have rejected whole genome duplications in vertebrates all together and only accept a continuous rate of gene duplication [15,16]. Recently, additional evidence for two rounds of whole genome duplications was presented [17], combining data from gene families, phylogenetic trees, and genomic map position. In particular, when examining the genomic map position of those genes in the human genome that can be traced back to a duplication event at the base of vertebrates, a clear pattern of tetra-paralogy emerges, making a convincing case for 1R/2R.

Whole genome duplication events shaping the genomes of vertebrates have not only been proposed in the early evolution of vertebrates, but also in the stem lineage of ray-finned (actinopterygian) fishes, after their divergence from the land vertebrates. Again, the first strong indications for a fish-specific genome duplication (FSGD) [18] came from studies based on *Hox *genes and *Hox *clusters. Extra *Hox *gene clusters discovered in the zebrafish (*Danio rerio*) [19], medaka (*Oryzias latipes*) [20], the African cichlid (*Oreochromis niloticus*) [21], and the pufferfish (*Takifugu rubripes*) [22], suggested an additional genome duplication in ray-finned fishes (Actinopterygii) before the divergence of most teleost species. Comparative genomic studies have also revealed many more genes and gene clusters for which two copies exist in teleost fishes but only one cognate copy in other vertebrates. The observations that different paralogs are found on different linkage groups and show synteny with other duplicated chromosomal regions [23] and that many paralogous pairs seem to have originated at about the same time [9,24] support the hypothesis that these genes arose through a complete genome duplication event during the evolution of the actinopterygian lineage. Both Vandepoele *et al*. [9] and Christoffels *et al*. [24] identified duplicated genes in *Takifugu *and estimated that 3R took place about 320 to 350 MYA. The split between ray-finned fishes and land vertebrates, dated at 450 MYA, was used as a calibration point for the dating of the gene duplication events in fishes. However, the most conclusive evidence for a complete genome duplication in ray-finned fishes came from the comparative analyses of the recently determined *Tetraodon *genome sequence and the human genome sequence [25]. Jaillon *et al*. [25] compared the chromosomal distribution of genes of *Tetraodon *with those in human and observed that many incidents of human synteny segments were found in duplicate on two different *Tetraodon *chromosomes.

Apart from a continuous creation of genes [26] through small-scale gene duplication events such as unequal crossing-over or reverse transcription, vertebrate genomes have thus most probably been shaped by two or more WGD events. As a matter of fact, it is even very tempting to speculate that vertebrates as we know them today might not have existed if it were not for these major duplication events [1,27,28]. Similarly, the fish-specific genome duplication might have contributed to the biological diversification of ray-finned fishes [18], although others reject such a hypothesis [29]. Nevertheless, a recent paper by Scannell *et al*. [30] suggests a clear link between genome duplication and speciation in yeasts, and also in plants, genome-wide duplication events have been associated with speciation and adaptive radiations [31-33].

Here, we report on gene gain and loss in seven different vertebrate genomes, namely human, mouse, rat, chicken, frog, zebrafish and pufferfish. The aims of our study were: to determine in which part of the vertebrate tree gene duplication and gene loss have been the most extensive; to investigate whether there is a bias in gene loss towards the functional class duplicated genes belong to and whether this is correlated with the mode of duplication (small-scale versus large-scale); and to speculate on the importance of these events for the evolution of vertebrates in general.

## Results and discussion

The current composition of vertebrate proteomes is, to a large extent, the result of gene duplication and gene loss events that have occurred at different times during vertebrate evolution [5,9,17] (see also this study). To study the consequences of these events on vertebrate proteomes and vertebrate evolution, we delineated gene families and used these for constructing phylogenetic trees (see Materials and methods). The delineation of gene families resulted in 9,461 families with a *Ciona *or *Drosophila *outgroup. As expected, *Ciona *was more often found as first outgroup sequence (better E-value score in BLAST) than *Drosophila *(5,609 versus 3,852, respectively). We discarded 602 multi-gene families, for which no *Ciona *or *Drosophila *outgroup sequence could be identified.

### Gene duplication in the vertebrate tree

**Figure 1:**
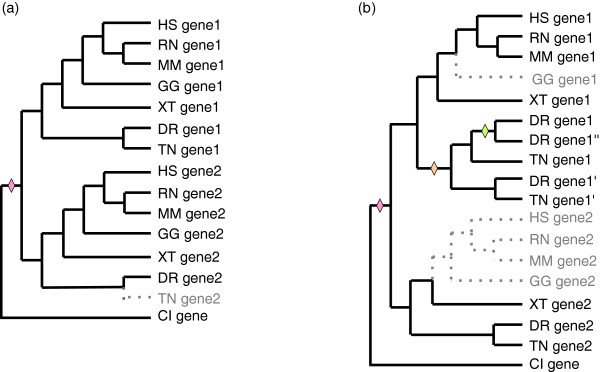
Hypothetical examples of phylogenetic trees with duplication and gene loss events. The phylogenetic trees were inferred from a gene family including members of all genomes used in the current study (human, *HS*; mouse, *MM*; rat, *RN*; chicken, *GG*; frog, *XT*; zebrafish, *DR*; *Tetraodon*, *TN*; *Ciona*, *CI*). All nodes are assumed to be supported by >70% in bootstrap analysis. Gene duplication can be recognized if at least two gene copies are present for the same species. **(a) **The duplication event (represented by a pink diamond) was inferred to have occurred early in vertebrate evolution because both land vertebrates and fishes have two copies of the gene. This is the most likely explanation, since the alternative assumption, where all lineages have undergone separate gene duplication events, is much less parsimonious. Subsequently, a gene loss event can be inferred for *Tetraodon*, since gene2 is missing (dotted line). The general conclusion of this hypothetical tree is thus one gene loss event of a *TN *duplicate that first had been created in the common ancestor of land vertebrates and fishes. For all other genomes, we count two retained duplicates after this ancient duplication event. **(b) **This more complex phylogenetic tree contains three duplication events (again indicated by diamonds). The oldest duplication event (pink diamond) is dated early in vertebrate evolution (TP13, similar to the one in (a)). *HS*, *MM*, *RN *and *GG *lost gene2, which is interpreted as gene loss of a TP13 duplicate in the common ancestor of these organisms (thus at TP7; Figure 2). *GG *also lost gene1, a gene loss event at TP6 of a duplicate that originated at TP13. The orange diamond indicates a duplication event in the common ancestor of the fishes, not shared with land vertebrates (TP12), resulting in gene1 and gene1' for both *DR *and *TN*. Finally, *DR *gene1 and gene1" are the result of a species-specific duplication event in *DR*.

**Figure 2:**
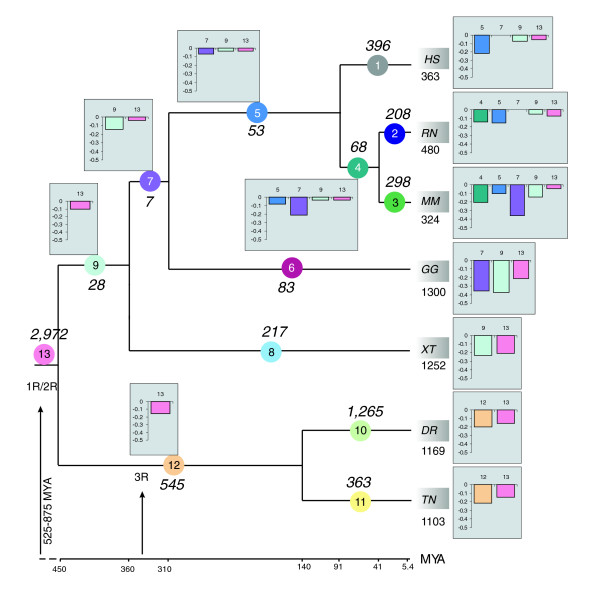
Gene duplications and gene losses mapped on the vertebrate tree. The vertebrate tree is shown with branch lengths proportional to time. The divergence times were taken from [35,68,69]. Abbreviations of species names are as in Figure 1. The numbers in colored circles indicate the different time points analyzed, referred to in the text as TPx. The total number of inferred duplications at each time point (TP) is shown in italics. The (negative) bars on the plots (with gray background) show the fraction of genes that was lost again after they have been created in a specific duplication event (indicated in colors corresponding to the time points (TP)). The total amount of gene loss for each organism is indicated under the species name.

On the basis of the 9,461 gene families, 8,165 phylogenetic trees containing 85,426 vertebrate genes were inferred, and speciation and duplication events were counted (59,852 and 11,167, respectively; Figure 1). The relative position of the duplicated genes in a vertebrate tree was used to determine timing of a duplication event. For ease of reference, the different branches in the tree, corresponding with certain periods in vertebrate evolution, are indicated by TPx, where x is a number between 1 and 13 (Figure 2). All trees can be consulted on our website [34]. The whole genome duplication (WGD) events during the early evolution of vertebrates (1R/2R) are assumed to have occurred before the divergence of the fishes and the tetrapods (indicated by TP13) [5,9]. TP12 marks the branch on which 3R (the FSGD) has occurred [18]. It should be noted though that, although we assume that many of the duplicates in TP13 and TP12 were created as a result of WGD events, a considerable fraction originated through small-scale duplication events, further referred to as the continuous mode of duplication.

As can be seen in Figure 2, the number of identified duplications at TP13 (2,972 duplications) exceeds all other values, which indeed suggests that gene duplication has been rampant during early vertebrate evolution. A high number of duplications is also detected in TP12 (branch of the FSGD). Nevertheless, the number of detected duplications in the common ancestor of the fishes (coinciding with 3R, TP12: 545 duplications) is more than five times smaller than the number of duplications on TP13. There might be several explanations for the large difference in the number of duplications between TP12 and TP13. First, the FSGD was one event (reviewed in [18]), while there is good evidence that there have been two WGD events at the dawn of vertebrate evolution (see, for example, [8,9,17]). Second, gene loss following the FSGD might have been more extensive than gene loss following 1R/2R (see below). In addition, a duplication on TP12 can only be recognized if at least one of the two fish (*Danio *or *Tetraodon*) has retained two copies of the same gene. Regarding the TP13 branch, there is a much greater chance to detect duplications, simply because there are more species in our dataset to compare with. As long as two duplicated genes that arose early in vertebrate evolution (for example, TP13) can be found in at least one of the extant vertebrate genomes, this can be regarded as evidence for a duplication event in the common ancestor of the vertebrates (TP13; see Materials and methods).

Apart from WGDs, gene duplication is a continuous process and, as expected, duplication events were found on all branches during vertebrate evolution. Nevertheless, there are remarkable differences in the number of duplications in the different branches. For instance, the number of zebrafish-specific gene duplicates seems exceptionally high, while that for *Tetraodon *is rather low. Zebrafish and *Tetraodon *are assumed to have diverged about 140 MYA [35], and since that time *Tetraodon *has retained 363 duplicates (TP11), while zebrafish counts almost four times as many duplicates (1,265, TP10). When the number of retained duplicates is divided by the time since speciation, a net average duplication rate of 9.04 duplications per million years in zebrafish is obtained, which is the highest of all lineages in the vertebrate tree, compared to only 2.6 for *Tetraodon*. Mouse and rat have values that lie in between those of zebrafish and *Tetraodon *(7.27 and 5.07 duplications per million years, respectively). Comparing these net rates to net rates in other terminal branches confirms the extremely high number of retained duplicates in zebrafish, rather than an exceptionally low rate in *Tetraodon*. The lower net rate of duplicate retention after species-specific duplications in rat (TP2) compared to mouse was already noted before [36]. The numbers of retained duplicates for frog and chicken are exceptionally low (0.6 in TP8, and 0.27 in TP6, respectively). It should be noted though that low values for duplications might reflect excessive gene loss in those lineages, rather than a decreased rate in gene duplication.

### Gene loss in the vertebrate tree

After a duplication event, the most likely fate of a duplicate is gene loss or nonfunctionalization [26,32]. However, there is a reasonable chance that both copies will be retained, after which different scenarios can be envisioned: one of the two duplicates might acquire a new function (neofunctionalization); the duplicates undergo so-called subfunctionalization, in which both gene copies lose a complementary set of subfunctions and thereby divide the ancestral gene's original functions [37], or instead of diverging in function both gene copies remain largely redundant and provide the organism with increased genetic robustness against harmful mutations [38]. Recent studies revealed that subfunctionalization can occur rapidly after duplication, and is often accompanied by neofunctionalization. This has led to a new model of gene function evolution called sub-neofunctionalization [39,40]. Shiu *et al*. [41] provided some evidence that positive selection plays a more important role in the retention of gene duplicates than subfunctionalization. A general overview of the mechanisms of gene duplication and retention can be found in [42,43].

In the current study, gene loss was determined as follows: when a sequence was missing from a certain species or a clade of species in a tree topology, while it was expected to be present because of a duplication event deeper in the same tree, this was counted as a gene loss event in the branch leading to the species or the clade (Figure 1). However, if one accepts that one or two WGD events have occurred in the early evolution of vertebrates (TP13), we can roughly estimate the amount of gene loss following these events. More in particular, we can infer where in the vertebrate tree these genes have been lost again. Since these WGDs originally duplicated all genes, we can assume that gene families for which such a duplication event at TP13 cannot be uncovered are characterized by an immediate gene loss of the duplicates created through 1R/2R. In the 7,350 gene families that have a representative in fish, land vertebrates and one of the outgroup sequences, 5,396 families were identified without a duplication on TP13, which seems to indicate massive gene loss following 1R/2R.

Similarly, fishes also seem to have lost many genes following the FSGD. As a matter of fact, both zebrafish and *Tetraodon *seem to have lost a similar amount of duplicates, although, as stated before, zebrafish has much more recent duplicates than *Tetraodon*. For instance, Figure 2 shows that both zebrafish and *Tetraodon *have lost about 30% of the genes that could still be identified as duplicated at TP13, about 15% before the split and about 15% after the split of both fish species (pink bins in plots, Figure 2). On top of that, about 20% of the duplicates resulting from the FSGD (or from small-scale duplication events that have occurred between 450 and 140 MYA; Figure 2) have been lost in both fish species.

Strikingly, all vertebrates continue to lose duplicates that were created at much earlier times. Figure 2 (pink bins in plots) clearly shows that duplicates that were created during 1R/2R can still be lost after they have survived for hundreds of millions of years of evolution. Some of those genes are lost only after the divergence of human and rodents, or after the divergence of mouse and rat. As a matter of fact, this is in congruence with what is predicted by age distributions of duplicated genes, which usually show an exponential or power-law decay of genes that have been duplicated [26,32], suggesting that, although chances become smaller, anciently duplicated genes are still getting lost. A high gene loss of such old duplicates is observed in particular for frog, chicken, and both fish genomes, while this is much less the case for mammals. Actually, gene loss in general is about four times higher in frog, chicken, and fishes, compared to primates (human) and rodents (mouse and rat). Extensive gene loss in the avian lineage has been reported before [44].

**Figure 3:**
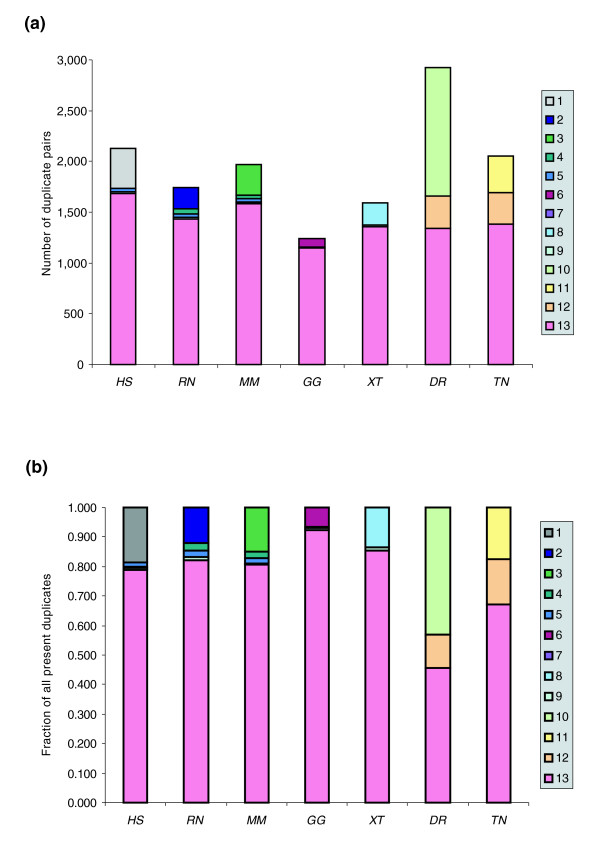
Origin of duplicates in different vertebrates. The number of duplicates and their origin (in the vertebrate tree) is shown for all organisms analyzed in the current study. **(a) **The absolute number of duplicated genes; **(b) **the relative contribution of the origin of duplicates to the total duplicate content of each vertebrate genome. Colors correspond to the duplication events indicated in Figure 2. Pink represents genes of which a major fraction is assumed to have been created during 1R/2R, while orange refers to the fraction of genes of which many are assumed to have been created during the FSGD.

We have also computed the relative contribution of gene duplication at different times in vertebrate evolution to the total gene or proteome content of extant vertebrates (Figure 3a). Rescaling to the fraction of retained duplicates of different origin versus the total number of retained duplicates (Figure 3b) provides a view on the relative importance of duplication events for the composition of current vertebrate proteomes. As can be seen in Figure 3a, the majority of duplicated genes in all vertebrate genomes have been created by ancient duplication events early in vertebrate evolution and coinciding with TP13 or 1R/2R (pink parts in graph of Figure 3). For fishes, the FSGD (TP12) has also contributed a considerable number of genes to their current genome content (orange parts in graphs of Figure 3). As can be observed, and stated above already, a majority of duplicates in zebrafish are of more recent origin (indicated in light green, TP10), created after the split between zebrafish and *Tetraodon*. Apparently, the zebrafish genome, unlike that of *Tetraodon*, has not only considerably expanded through the accumulation of (retro-) transposable elements [45], but also through a large number of recent duplications.

As can be seen in Figure 3, the gene duplicates of ancient origin (TP13) contribute considerably less to the whole of the current paranome (the full set of paralogs in a genome) of fishes than to that of the land vertebrates, because of both the FSGD and species specific duplications. Indeed, while for land vertebrates the fraction of the paranome formed by ancient duplicates amounts to 80% or more, this is only 50% for zebrafish and 70% for *Tetraodon*. However, the total number of ancient duplicates in fish genomes is still very similar to that of chicken and frog, and only slightly less than in human or rodents.

### Which genes have been retained or lost?

To determine whether gene families involved in different biological processes or with distinct biochemical functions show dissimilar patterns of gene retention and gene loss, the Gene Ontology (GO) controlled vocabulary was used [46] (see Materials and methods). In total, 7,314 trees with GOslim annotation could be analyzed (Table 1).

In a first step, we compared the paranome of all organisms in a pairwise manner, without considering the mode and time of origin of the duplicates. Several functional categories with a significantly different number of retained duplicates could be identified. Interestingly, all of the significant differences were observed between fishes on the one hand and land vertebrates on the other (Additional data file 1, Table S1). For instance, genes belonging to the GOslim category 'catalytic activity' have been retained in excess in both zebrafish and *Tetraodon *compared to the land vertebrates, whereas other categories such as 'protein modification', 'protein metabolism', 'catabolism', and 'peptidase activity' show the opposite trend (Additional data file 1; Table S1). Significant differences regarding gene retention for different functional categories within the land vertebrates or within the fishes could not be observed, suggesting that land vertebrates on the one hand and fishes on the other show similar gene loss for the same functional classes of genes.

In a second step, we investigated the effect of the time and mode of duplication on the retention of genes belonging to different functional categories. We already showed that the WGDs have played a major role in contributing extra genes to vertebrate genomes. In addition, there seems to be a considerable functional bias in gene retention following these major events (1R/2R, TP13 and 3R, TP12). For instance, for most vertebrates, genes belonging to GO classes such as 'protein binding', 'signal transduction', 'transcription', 'development', 'DNA binding', 'receptor activity', 'ion transport', and 'protein modification' show significantly higher levels of gene retention following WGD events (TP12 and TP13; Table 2) than when such genes are being created in smaller-scale events. Again, it should be noted that we assume that many of the genes created at TP12 and TP13 are the result of a WGD event (see above). On the other hand, genes belonging to other functional classes such as 'electron transport', 'amino acid and derivative metabolism' and 'RNA binding' seem to have been retained in particular following small-scale duplication events in at least four different species of the dataset (Table 2). The strong bias in gene retention for regulatory genes following large-scale gene duplication events is very much in congruence with what has been observed in plants. There too, genes involved in transcriptional regulation and signal transduction seem to have been preferentially retained following genome duplications [32,47,48]. Even in yeast, a large number of duplicates resulting from the WGD with functions in transcription were observed [49]. Similarly, in both vertebrates (this study) and plants [32,33], developmental genes are observed to be well retained following genome duplications. Furthermore, the high retention rate of transcription factors following WGD events might be explained by the fact that they are often active as protein complexes and probably need to be present in stoichiometric quantities for their correct functioning. This is also supported by the retention of genes belonging to classes such as 'protein binding' and 'protein modification' following WGDs. As a matter of fact, the higher retention of genes belonging to these particular classes is predicted by the 'gene balance' hypothesis, which states that retention of genes that may have strong dosage effects, such as transcription factors, will be selected against if they are copied without their partners in the regulatory or protein interaction network [50-53]. On the other hand, if the genes, encoding products that cooperate in the same complex pathway or network, are duplicated at the same time, which is the case in WGDs, gene dosage effects might be avoided by retaining all genes in that particular complex or network. It should be noted that the exceptionally high number of species-specific duplications in zebrafish outshines the number of retained WGD duplicates in this proteome. This explains why many genes of GOs resulting from WGDs are retained in excess in all species except zebrafish (Table 2).

**Figure 4:**
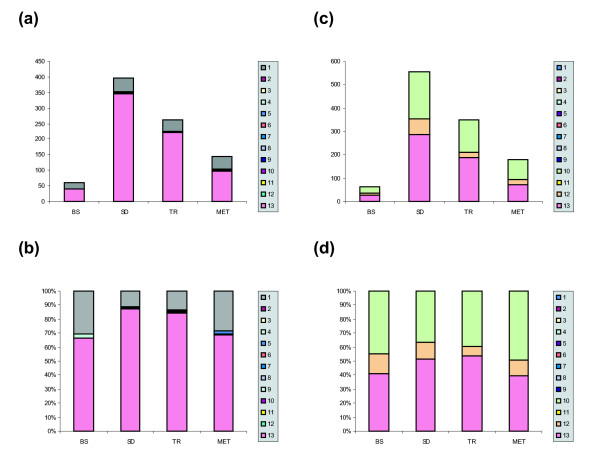
Retention of duplicates in human and zebrafish following WGDs and small-scale duplications for four different functional categories. The retention of duplicates in **(a,b) **human and **(c,d) **zebrafish following WGD events (assumed at TP12 and TP13) versus small-scale duplication events for the GOSlim ontologies 'biotic stimulus' (BS), 'signal transduction' (SD), 'transcription' (TR), and 'metabolism' (MET). Color codings correspond to time points in Figure 2. (a,c) Absolute numbers of retained duplicates. (b,d) Relative numbers of retained duplicates normalized for the total amount of duplicates in the genome.

Figure 4 shows the retention of duplicates in human and zebrafish following WGD events (assumed at TP12 and TP13) versus small-scale duplication events for the GOSlim ontologies 'biotic stimulus, 'signal transduction', 'transcription', and 'metabolism'. As can be seen, apart from a larger number of genes that have to do with signal transduction and transcription factor activity, these genes are also retained at a higher level following WGD events. Compared to these genes, the contribution of small-scale duplication events to the retention of genes involved in 'biotic stimulus' and 'metabolism' is much more significant, in particular for human. As a matter of fact, duplicates involved in 'response to biotic stimulus' occur all throughout the vertebrate tree, and none of the branches in the vertebrate tree contain exceptionally few or many duplicates. For instance, the amount of gene loss following 1R/2R was not significantly higher or lower than expected. This again confirms previous findings that genes involved in secondary metabolism or response to biotic stimuli tend to be preserved regardless of the mode of duplication because they are important adaptive traits that are heavily selected for during evolution [32].

## Conclusion

It has been shown before that gene duplication greatly contributed to the complexity of eukaryotic genomes [54,55]. Although gene and genome duplication events in vertebrates might have been less extensive than in plants [56], in vertebrates a major part of the proteome also consists of proteins encoded by duplicated genes. As shown previously, many of the duplicates residing in vertebrate genomes have been created during very ancient paleopolyploidy events [5,9,17]. As previously observed for plants [32,33,47,48], in vertebrates there also seems to be a significant difference in gene retention between genes created in polyploidy events or in small-scale duplication events. In addition, similar to plants, and as predicted by the 'gene-balance' hypothesis [51-53], retention for genes encoding proteins that are active as protein complexes and need to be present in stoichiometric quantities for their correct functioning (for example, genes involved in transcriptional regulation and signal transduction), is high following (paleo)polyploidy events, while this is not the case when such genes are being duplicated individually.

It has been suggested that polyploidy events can be associated with important evolutionary transitions, major leaps in development, and/or adaptive radiations of species [1,18,28,31,33,53,57]. The high retention of many important genes involved in transcriptional regulation, signaling, and development after paleopolyploidy events as shown here and in previous studies, and the fact that in particular such genes are considered important for introducing phenotypic variation and increase in biological complexity, makes it tempting to speculate on the importance of such large-scale duplication events for vertebrate evolution. As far as we know, within the animal lineage, vertebrate genomes are still the most complex, and, at least compared to invertebrates, vertebrates still do contain considerably more genes, a majority of which is probably created by gene and ancient genome duplications. Whether these 'ancient' extra genes are sufficient to explain the increased morphological complexity of vertebrates is doubtful, but they might hold at least part of the answer.

## Materials and methods

### Construction of the dataset and phylogenetic analysis of gene families

The predicted protein sequences from human (release 31.35d), mouse (release 31.33g), *Tetraodon nigroviridis *(release 31.1c), zebrafish (*Danio rerio*, release Zfish5), rat (*Rattus norvegicus*, release 31.34a), chicken (*Gallus gallus*, release 31.1g), and frog (*Xenopus tropicalis*, release 31.1a) were retrieved from Ensembl [58]. If multiple splice variants were reported for one gene, only the longest transcript was used. Transposon-like genes were removed based on homology with known transposons [59].

To delineate vertebrate gene families, a similarity search was performed (BLASTP, [60]; E-value cutoff E-10) with all proteins from the organisms listed above, plus the proteins of *Ciona intestinalis *([61], version 1) and *Drosophila melanogaster *(Ensembl [58], version 3), which were added as outgroup species. Because the focus of this study was to identify genes that were duplicated during vertebrate evolution, only vertebrate genes were used as blast query. Blast hits between vertebrate sequences with a better score than the best score between a vertebrate sequence and an outgroup sequence (*Drosophila *or *Ciona*) were retained in the gene family. The *Drosophila *or *Ciona *sequence was used to root the phylogenetic tree (see below). Redundancy between the families was removed. Gene families without a homolog in *Ciona *or *Drosophila *were discarded from the dataset.

For all retained gene families a multiple alignment was created with T-Coffee 1.37 using default parameters [62]. Alignment columns containing gaps were removed when a gap was present in >10% of the sequences. To reduce the chance of including misaligned amino acids, all positions in the alignment left or right from the gap were also removed until a column in the sequence alignment was found where the residues were conserved in all genes included in our analyses. This was determined as follows: for every pair of residues in the column, the BLOSUM62 value was retrieved. If at least half of the pairs had a BLOSUM62 value ≥0, the column was considered as conserved.

Neighbor joining trees (with 500 bootstrap replicates) were constructed using PHYLIP 3.5 [63]. Families containing more than 100 proteins were aligned with ClustalW 1.83 [64] and were bootstrapped 100 times. Families with more than 260 genes were excluded from the dataset, because PHYLIP was unable to build a phylogenetic tree. The neighbor-joining trees were parsed, and phylogenetic information supported by a bootstrap value of at least 70% was considered for further analysis.

### Functional annotation of the gene families

Gene families were functionally annotated using GO [46,65]. The GO annotation for human and mouse, as well as the InterPro annotation for all organisms listed above was downloaded from Ensembl. For the organisms that did not have a GO annotation, a GO labeling was obtained based on the InterPro annotation [66]. In a first step, the InterPro annotation was linked to the corresponding GO annotation with the InterPro2GO mapping ([65], version of April 2005). Since some GO categories only contain a small fraction of genes, in a second step all GO labels were remapped to the GOslim ontology, a reduced version of GO ontology ([65], generic version of August 2002). GO annotation per family was obtained by listing the GOslim labels for all the genes of that family. A weight, equal to the percentage of genes with GOslim annotation within the same subcategory (molecular function, cellular component, biological process) that carried this label, was attached to all the GOslim labels. Only GOslim labels with a weight greater than 30% were considered as representative for the family because it is unlikely that a GOslim label ascribing fewer members of that family is representative for the entire family. A lower cutoff leads to a considerable increase in the number of GOslim labels for each family, which means that rare GO labels would be assigned to an entire family. A higher cutoff only decreased the number of GOslim labels for each family slightly (Additional data file 1; Figure S1).

### Relative dating of duplication events

Phylogenetic trees were systematically analyzed for the presence of gene duplication events at different points in vertebrate evolution (see below). Duplication events were evaluated by relative dating, thus based on the relative position of the duplicated genes compared to speciation events in the phylogenetic tree. Gene loss following gene duplication events was always counted as parsimonious as possible. Two hypothetical examples that explain identification of gene loss after duplication are given in Figure 1. For instance, a duplication event is registered at TP13 (Figure 2) if a land vertebrate and a fish gene are clustered at one side of the root and at least one other vertebrate sequence is found on the other side of the root. For example, the topology (((TNgene1, HSgene1), HSgene2), CI), supported by bootstrap values higher than 70%, is a minimal requirement for accepting a duplication event at TP13. A tree with only mammals and amphibians, such as (((HSgene1, XTgene1), (HSgene2, XTgene2)), CI) does contain a duplication event, shared by mammals and amphibians, but does not allow one to correctly identify the time point of the duplication event since it is possible that the duplication took place at TP9 or at TP13. To identify a duplication on TP9, we need a fish gene that clusters outside the duplication node. All scripts to parse phylogenetic trees are available upon request.

To determine if there are any significant differences in the total number of duplicates in the vertebrate proteomes, we performed pairwise comparisons and used the Fisher's exact test. This test was also used to determine whether gene retention at different time points or between different species is biased towards particular functional categories (GOslim) [34]. The false discovery rate method (q-value) [67] was used to correct for multiple hypotheses testing and adjusted *p* values smaller than 0.05 were considered as significant.

## Additional data files

The following additional data are available with the online version of this paper. Additional data file 1 contains additional information about the applied methods and the results, including: an explanation about the setting of the weight cut-off in the labelling of gene families with GOslim annotation; Table S1, showing significant trends in the total amount of duplicate pairs in the vertebrate genomes; and Table S2, showing the excess of gene retention in parts of the vertebrate tree. Additional data file 2 lists the proteins with descriptions (Ensembl).

## Authors' contributions

T.B. designed the research, analyzed data and wrote the paper. K.V. helped in research design and writing the paper. S.D.B. provided technical assistance and scientific guidance. C.S. provided technical assistance. S.M. provided scientific guidance. Y.V.d.P. designed the research, supervised the project, and wrote the paper.

## Supplementary Material

Additional File 1 Contains an explanation about the setting of the weight cutoff in the labelling of gene families with GOslim annotation; Table S1, showing significant trends in the total amount of duplicate pairs in the vertebrate genomes; and Table S2, showing the excess of gene retention in parts of the vertebrate tree. Click here for file

Additional File 2 Proteins with descriptions (Ensembl). Click here for file

## Figures and Tables

**Table 1:** Number of genes in genomes, gene families, phylogenetic trees, and trees with GOslim annotation

	*Homo sapiens*		*Rattus norvegicus*		*Mus musculus*		*Gallus gallus*		*Xenopus tropicalis*		*Danio rerio*		*Tetraodon nigroviridis*	
Genome	22,218		21,952		24,461		17,709		24,405		22,877		28,005	
Gene families	14,054	(0.63)	14,155	(0.64)	14,813	(0.61)	9,875	(0.56)	13,336	(0.55)	14,597	(0.64)	12,373	(0.44)
Phylogenetic trees	13,080	(0.59)	12,537	(0.57)	13,325	(0.54)	9,292	(0.52)	11,747	(0.48)	13,785	(0.60)	11,660	(0.42)
Phylogenetic trees with GO annotation	12,470	(0.56)	11,919	(0.54)	12,669	(0.52)	8,806	(0.50)	11,049	(0.45)	13,244	(0.58)	11,097	(0.40)

The fraction of the proteome analyzed at a certain step in the procedure is in parentheses (see Materials and methods for details).

**Table 2:** Excess of gene retention in parts of the vertebrate tree

GOslim label, category, description	Organism	TPs showing significant difference	TP with highest number of duplicates	q-value
GO:0006118, BP, electron transport	*HS*	TP13 vs TP1	TP1	6.73E-05
	*DR*	TP12 vs TP10	TP10	2.50E-04
	*DR*	TP13 vs TP10	TP10	8.60E-06
GO:0006519, BP, amino acid and derivative metabolism	*HS*	TP13 vs TP1	TP1	8.69E-04
	*DR*	TP12 vs TP10	TP10	5.37E-04
	*DR*	TP13 vs TP10	TP10	1.47E-08
GO:0007165, BP, signal transduction	*TN*	TP13 vs TP11	TP13	5.76E-04
	*XT*	TP13 vs TP8	TP13	9.18E-04
	*RN*	TP13 vs TP2	TP13	1.44E-17
	*MM*	TP13 vs TP3	TP13	8.32E-19
	*HS*	TP13 vs TP1	TP13	2.43E-02
	*DR*	TP12 vs TP10	TP10	3.60E-16
	*DR*	TP13 vs TP10	TP10	6.40E-12
GO:0003677, MF, DNA binding	*XT*	TP13 vs TP8	TP13	4.38E-02
	*RN*	TP13 vs TP2	TP13	1.20E-08
	*MM*	TP13 vs TP3	TP13	1.86E-07
	*DR*	TP12 vs TP10	TP10	1.51E-10
	*DR*	TP13 vs TP10	TP10	3.99E-10
GO:0004872, MF, receptor activity	*TN*	TP13 vs TP11	TP13	6.34E-03
	*XT*	TP13 vs TP8	TP13	5.83E-03
	*RN*	TP13 vs TP2	TP13	2.26E-11
	*MM*	TP13 vs TP3	TP13	7.47E-09
	*DR*	TP12 vs TP10	TP10	1.01E-08
	*DR*	TP13 vs TP10	TP10	7.37E-05
GO:0006464, BP, protein modification	*RN*	TP13 vs TP2	TP13	8.16E-12
	*MM*	TP13 vs TP3	TP13	7.26E-07
	*HS*	TP13 vs TP1	TP13	2.43E-02
	*DR*	TP12 vs TP10	TP10	1.00E-09
	*DR*	TP13 vs TP10	TP10	2.63E-07
GO:0005515, MF, protein binding	*TN*	TP13 vs TP11	TP13	5.76E-04
	*XT*	TP13 vs TP8	TP13	1.01E-02
	*RN*	TP13 vs TP2	TP13	2.31E-12
	*MM*	TP13 vs TP3	TP13	3.72E-08
	*DR*	TP12 vs TP10	TP10	7.78E-07
	*DR*	TP13 vs TP10	TP10	4.01E-06
GO:0007275, BP, development	*TN*	TP13 vs TP11	TP13	3.71E-02
	*XT*	TP13 vs TP8	TP13	4.50E-02
	*RN*	TP13 vs TP2	TP13	7.64E-08
	*MM*	TP13 vs TP3	TP13	1.49E-06
	*HS*	TP13 vs TP1	TP13	1.88E-02
	*DR*	TP12 vs TP10	TP10	1.18E-06
	*DR*	TP13 vs TP10	TP10	1.06E-03
GO:0006350, BP, transcription	*TN*	TP13 vs TP11	TP13	1.97E-02
	*RN*	TP13 vs TP2	TP13	7.13E-11
	*MM*	TP13 vs TP3	TP13	3.82E-11
	*DR*	TP12 vs TP10	TP10	1.54E-20
	*DR*	TP13 vs TP10	TP10	4.11E-11
GO:0003723, MF, RNA binding	*RN*	TP13 vs TP2	TP2	4.71E-02
	*HS*	TP13 vs TP1	TP1	9.08E-03
	*DR*	TP12 vs TP10	TP10	9.36E-04
	*DR*	TP13 vs TP10	TP10	3.42E-05
GO:0006811, BP, ion transport	*TN*	TP13 vs TP11	TP13	1.11E-02
	*XT*	TP13 vs TP8	TP13	1.79E-02
	*RN*	TP13 vs TP2	TP13	1.23E-11
	*MM*	TP13 vs TP3	TP13	1.34E-06
	*HS*	TP13 vs TP1	TP13	3.84E-02
	*DR*	TP12 vs TP10	TP10	8.52E-03
	*DR*	TP13 vs TP10	TP10	1.00E-02

The GOslim label, its category (MF, molecular function; BP, biological process) and the general description are shown. For each organism (*HS*, human; *MM*, mouse; *RN*, rat; *GG*, chicken; *XT*, frog; *DR*, zebrafish; *TN*, *Tetraodon*), the number of species-specific duplicates was compared to the number of duplicates from time points (TPs) coinciding with WGDs (TP12 and TP13). The time points showing a significant difference (q < 0.05) in comparison are shown (TPx vs TPy), followed by the time point with the highest number of duplicates. The last column shows the q-value. Only significant results that are discussed in the text are listed (others can be found in Additional data file 1; Table S2).
