# Supplementary material for: The gain and loss of genes during 600 million years of vertebrate evolution
Source: Genome Biol. 2006 May 24;7(5):R43. doi: 10.1186/gb-2006-7-5-r43 (PMC1779523; doi:10.1186/gb-2006-7-5-r43)
Supplement: Additional File 1 — Contains an explanation about the setting of the weight cutoff in the labelling of gene families with GOslim annotation; Table S1, showing significant trends in the total amount of duplicate pairs in the vertebrate genomes; and Table S2, showing the excess of gene retention in parts of the vertebrate tree. [file gb-2006-7-5-r43-S1.doc]

Supplementary material

Setting the weight cut-off in the labelling of gene families with GOslim annotation

In a first step, all GOslim labels were collected for all genes in the families. The labels were subdivided by functional category (molecular function - MF, biological process - BP, cellular component - CC). The cellular component labels were discarded. For each label, a weight was calculated: the number of genes with the GOslim label in the family was divided by the number of genes with GOslim annotation within the functional category (e.g. molecular function) of the GOslim label. Subsequently, a minimum weight was used in order to avoid that gene families were annotated by rare labels. Figure S1 shows several possible weight cut-off values in the abscis. The plots show how many gene labels were appointed to the family. The values on the ordinate show the frequency (number of gene families) of that number of gene labels.

Figure S1 shows that if the cut-off value increases, the amount of gene families with many GOslim labels decreases (the peaks get sharper and go to the left).

Finally, it was decided to choose cut-off value 0.3, since this is the first weight cut-off without the strong tail on the right side of the peak, and without any family without GOslim annotation (the blue bar 0 is absent).

**Figure S1. Comparison between different weight cut-off values in the functional annotation of gene families**

Supplementary tables

**Table S1. Significant trends in the total amount of duplicate pairs in the vertebrate genomes**

The total amount of retained duplicates in the genome for a particular function was compared between all organisms, and significant results are listed. The genome (human - HS, mouse - *MM*, rat - *RN*, chicken - *GG*, frog - *XT*, zebrafish - *DR*, *Tetraodon*  - *TN*), with the significantly higher amount of duplicates is given in the fifth column.

| points | GOlabel | subtype | description | highest | p-value | q-value |
| --- | --- | --- | --- | --- | --- | --- |
| HS versus *DR* | GO:0003824 | MF | catalytic activity | *DR* | 4.02E-06 | 0.000362 |
| HS versus *DR* | GO:0006810 | BP | transport | HS | 0.000762 | 0.034283 |
| *RN* versus *DR* | GO:0008233 | MF | peptidase activity | *RN* | 0.003331 | 0.041688 |
| *RN* versus *DR* | GO:0003824 | MF | catalytic activity | *DR* | 8.59E-07 | 7.73E-05 |
| *RN* versus *DR* | GO:0005488 | MF | binding | *RN* | 0.001509 | 0.041688 |
| *RN* versus *DR* | GO:0005216 | MF | ion channel activity | *RN* | 0.005095 | 0.041688 |
| *RN* versus *DR* | GO:0019538 | BP | protein metabolism | *RN* | 0.007502 | 0.048229 |
| *RN* versus *DR* | GO:0006629 | BP | lipid metabolism | *RN* | 0.003545 | 0.041688 |
| *RN* versus *DR* | GO:0006810 | BP | transport | *RN* | 0.000118 | 0.005316 |
| *RN* versus *DR* | GO:0006464 | BP | protein modification | *RN* | 0.006808 | 0.048229 |
| *RN* versus *DR* | GO:0006259 | BP | DNA metabolism | *RN* | 0.007069 | 0.048229 |
| *RN* versus *DR* | GO:0005515 | MF | protein binding | *RN* | 0.003525 | 0.041688 |
| *RN* versus *DR* | GO:0007582 | BP | physiological process | *RN* | 0.004757 | 0.041688 |
| *RN* versus *DR* | GO:0007010 | BP | cytoskeleton organization and biogenesis | *RN* | 0.004819 | 0.041688 |
| *RN* versus *DR* | GO:0005215 | MF | transporter activity | *RN* | 0.004672 | 0.041688 |
| *RN* versus *DR* | GO:0009056 | BP | catabolism | *RN* | 0.003757 | 0.041688 |
| *MM* versus *DR* | GO:0008233 | MF | peptidase activity | *MM* | 0.000462 | 0.008351 |
| *MM* versus *DR* | GO:0003824 | MF | catalytic activity | *DR* | 2.03E-07 | 1.83E-05 |
| *MM* versus *DR* | GO:0005488 | MF | binding | *MM* | 0.005093 | 0.044283 |
| *MM* versus *DR* | GO:0019538 | BP | protein metabolism | *MM* | 0.000452 | 0.008351 |
| *MM* versus *DR* | GO:0006629 | BP | lipid metabolism | *MM* | 0.003798 | 0.042725 |
| *MM* versus *DR* | GO:0006810 | BP | transport | *MM* | 0.000464 | 0.008351 |
| *MM* versus *DR* | GO:0006464 | BP | protein modification | *MM* | 0.003279 | 0.042162 |
| *MM* versus *DR* | GO:0005515 | MF | protein binding | *MM* | 0.004341 | 0.043409 |
| *MM* versus *DR* | GO:0005215 | MF | transporter activity | *MM* | 0.003033 | 0.042162 |
| *MM* versus *DR* | GO:0006811 | BP | ion transport | *MM* | 0.005412 | 0.044283 |
| *MM* versus *DR* | GO:0009056 | BP | catabolism | *MM* | 0.000439 | 0.008351 |
| *MM* versus *TN* | GO:0008233 | MF | peptidase activity | *MM* | 0.000548 | 0.024664 |
| *MM* versus *TN* | GO:0003824 | MF | catalytic activity | *TN* | 0.000904 | 0.027113 |
| *MM* versus *TN* | GO:0019538 | BP | protein metabolism | *MM* | 8.43E-05 | 0.007587 |
| *GG* versus *DR* | GO:0003824 | MF | catalytic activity | *DR* | 1.36E-05 | 0.001224 |
| *GG* versus *DR* | GO:0005216 | MF | ion channel activity | *GG* | 0.002726 | 0.049063 |
| *GG* versus *DR* | GO:0000166 | MF | nucleotide binding | *GG* | 0.001225 | 0.028486 |
| *GG* versus *DR* | GO:0006810 | BP | transport | *GG* | 3.58E-05 | 0.001613 |
| *GG* versus *DR* | GO:0006464 | BP | protein modification | *GG* | 0.001266 | 0.028486 |
| *XT* versus *DR* | GO:0008233 | MF | peptidase activity | *XT* | 0.002871 | 0.043063 |
| *XT* versus *DR* | GO:0003824 | MF | catalytic activity | *DR* | 3.67E-05 | 0.00165 |
| *XT* versus *DR* | GO:0005488 | MF | binding | *XT* | 0.000161 | 0.004815 |
| *XT* versus *DR* | GO:0019538 | BP | protein metabolism | *XT* | 0.000419 | 0.00942 |
| *XT* versus *DR* | GO:0006810 | BP | transport | *XT* | 0.001666 | 0.029989 |
| *XT* versus *DR* | GO:0009056 | BP | catabolism | *XT* | 2.15E-05 | 0.00165 |
| *XT* versus *TN* | GO:0019538 | BP | protein metabolism | *XT* | 8.05E-05 | 0.007249 |
| *XT* versus *TN* | GO:0009056 | BP | catabolism | *XT* | 0.000278 | 0.012502 |

**Table S2. Excess of gene retention in parts of the vertebrate tree.**

The GOslim label, its category (molecular function and biological process) and the general description are shown. For each organism, the number of species-specific duplicates was compared to the number of duplicates from time points coinciding with WGDs (TP12 and TP13). For the species-specific duplications, the number of genes that were duplicated in a terminal branch was compared to the number of genes of that species in the same tree topology, but without species-specific duplication. For the time points coinciding with WGDs, the number of retained duplicate pairs after these duplications was compared to an estimation (based on tree topology) of the number of genes that were duplicated, but not retained.

The time points that were significantly different (q < 0.05) in comparison are shown (TPx vs TPy), followed by the time point with the highest number of duplicates. The last column shows the q-value. Only discussed significant results were listed in Table 2, this table shows all results.

| GO:0008233 | MF | peptidase activity | |
| --- | --- | --- | --- |
| *RN* | TP13 vs TP2 | TP13 | 9.82E-03 |
| *MM* | TP13 vs TP3 | TP13 | 1.08E-02 |
| *DR* | TP12 vs TP10 | TP10 | 2.48E-05 |
| *DR* | TP13 vs TP10 | TP10 | 5.55E-08 |
|  |  |  |  |
| GO:0003774 | MF | motor activity | |
| *RN* | TP13 vs TP2 | TP13 | 1.28E-02 |
| *MM* | TP13 vs TP3 | TP13 | 1.25E-02 |
|  |  |  |  |
| GO:0006118 | BP | electron transport | |
| HS | TP13 vs TP1 | TP1 | 6.73E-05 |
| *DR* | TP12 vs TP10 | TP10 | 2.50E-04 |
| *DR* | TP13 vs TP10 | TP10 | 8.60E-06 |
|  |  |  |  |
| GO:0009058 | BP | biosynthesis | |
| *RN* | TP13 vs TP2 | TP13 | 4.03E-03 |
| *MM* | TP13 vs TP3 | TP13 | 1.73E-02 |
| HS | TP13 vs TP1 | TP1 | 2.43E-02 |
| *DR* | TP12 vs TP10 | TP10 | 1.29E-05 |
| *DR* | TP13 vs TP10 | TP10 | 1.34E-08 |
|  |  |  |  |
| GO:0004672 | MF | protein kinase activity | |
| *RN* | TP13 vs TP2 | TP13 | 7.43E-05 |
| *MM* | TP13 vs TP3 | TP13 | 1.32E-03 |
| HS | TP13 vs TP1 | TP13 | 6.20E-03 |
| *DR* | TP12 vs TP10 | TP10 | 5.55E-04 |
| *DR* | TP13 vs TP10 | TP10 | 7.28E-03 |
|  |  |  |  |
| GO:0007028 | BP | cytoplasm organization and biogenesis | |
| *DR* | TP13 vs TP10 | TP10 | 1.15E-04 |
|  |  |  |  |
| GO:0004871 | MF | signal transducer activity | |
| *RN* | TP13 vs TP2 | TP13 | 2.69E-03 |
| *MM* | TP13 vs TP3 | TP13 | 1.90E-04 |
| *DR* | TP12 vs TP10 | TP10 | 5.37E-04 |
| *DR* | TP13 vs TP10 | TP10 | 2.11E-03 |
|  |  |  |  |
| GO:0015031 | BP | protein transport | |
| *MM* | TP13 vs TP3 | TP13 | 3.72E-03 |
| *DR* | TP12 vs TP10 | TP10 | 5.34E-04 |
| *DR* | TP13 vs TP10 | TP10 | 7.68E-04 |
|  |  |  |  |
| GO:0006519 | BP | amino acid and derivative metabolism | |
| HS | TP13 vs TP1 | TP1 | 8.69E-04 |
| *DR* | TP12 vs TP10 | TP10 | 5.37E-04 |
| *DR* | TP13 vs TP10 | TP10 | 1.47E-08 |
|  |  |  |  |
| GO:0009607 | BP | response to biotic stimulus | |
| *XT* | TP13 vs TP8 | TP8 | 4.38E-02 |
| HS | TP13 vs TP1 | TP1 | 2.43E-02 |
| *DR* | TP13 vs TP10 | TP10 | 9.03E-03 |
|  |  |  |  |
| GO:0007610 | BP | behavior | |
| *RN* | TP13 vs TP2 | TP13 | 2.73E-03 |
| *MM* | TP13 vs TP3 | TP13 | 2.98E-04 |
| *DR* | TP12 vs TP10 | TP10 | 3.19E-02 |
|  |  |  |  |
| GO:0009605 | BP | response to e*XT*e*RN*al stimulus | |
| *MM* | TP13 vs TP3 | TP13 | 1.45E-02 |
| *DR* | TP12 vs TP10 | TP10 | 1.41E-02 |
| *DR* | TP13 vs TP10 | TP10 | 9.15E-04 |
|  |  |  |  |
| GO:0007165 | BP | signal transduction | |
| *TN* | TP13 vs TP11 | TP13 | 5.76E-04 |
| *XT* | TP13 vs TP8 | TP13 | 9.18E-04 |
| *RN* | TP13 vs TP2 | TP13 | 1.44E-17 |
| *MM* | TP13 vs TP3 | TP13 | 8.32E-19 |
| HS | TP13 vs TP1 | TP13 | 2.43E-02 |
| *DR* | TP12 vs TP10 | TP10 | 3.60E-16 |
| *DR* | TP13 vs TP10 | TP10 | 6.40E-12 |
|  |  |  |  |
| GO:0008152 | BP | metabolism | |
| *RN* | TP13 vs TP2 | TP13 | 1.20E-03 |
| *MM* | TP13 vs TP3 | TP13 | 8.18E-03 |
| HS | TP13 vs TP1 | TP1 | 8.69E-04 |
| *DR* | TP12 vs TP10 | TP10 | 1.25E-08 |
| *DR* | TP13 vs TP10 | TP10 | 5.01E-15 |
|  |  |  |  |
| GO:0008092 | MF | cytoskeletal protein binding | |
| *RN* | TP13 vs TP2 | TP13 | 4.09E-02 |
| *DR* | TP12 vs TP10 | TP10 | 2.88E-02 |
| *DR* | TP13 vs TP10 | TP10 | 3.66E-02 |
|  |  |  |  |
| GO:0006091 | BP | generation of precursor metabolites and energy | |
| *DR* | TP12 vs TP10 | TP10 | 3.19E-02 |
| *DR* | TP13 vs TP10 | TP10 | 9.07E-06 |
|  |  |  |  |
| GO:0007154 | BP | cell co*MM*unication | |
| *RN* | TP13 vs TP2 | TP13 | 9.13E-06 |
| *MM* | TP13 vs TP3 | TP13 | 5.21E-06 |
| HS | TP13 vs TP1 | TP13 | 2.08E-02 |
| *DR* | TP12 vs TP10 | TP10 | 1.92E-04 |
| *DR* | TP13 vs TP10 | TP10 | 3.44E-04 |
|  |  |  |  |
| GO:0003824 | MF | catalytic activity | |
| *RN* | TP13 vs TP2 | TP13 | 6.24E-08 |
| *MM* | TP13 vs TP3 | TP13 | 6.47E-10 |
| HS | TP13 vs TP1 | TP1 | 2.78E-04 |
| *DR* | TP12 vs TP10 | TP10 | 9.00E-29 |
| *DR* | TP13 vs TP10 | TP10 | 3.34E-53 |
|  |  |  |  |
| GO:0005488 | MF | binding | |
| *TN* | TP13 vs TP11 | TP13 | 2.55E-02 |
| *XT* | TP13 vs TP8 | TP13 | 3.05E-02 |
| *RN* | TP13 vs TP2 | TP13 | 6.25E-09 |
| *MM* | TP13 vs TP3 | TP13 | 3.01E-09 |
| *DR* | TP12 vs TP10 | TP10 | 3.65E-13 |
| *DR* | TP13 vs TP10 | TP10 | 6.12E-13 |
|  |  |  |  |
| GO:0009719 | BP | response to endogenous stimulus | |
| HS | TP13 vs TP1 | TP1 | 3.70E-02 |
| *DR* | TP13 vs TP10 | TP10 | 1.00E-02 |
|  |  |  |  |
| GO:0005216 | MF | ion channel activity | |
| *TN* | TP13 vs TP11 | TP13 | 2.03E-02 |
| *RN* | TP13 vs TP2 | TP13 | 9.03E-10 |
| *MM* | TP13 vs TP3 | TP13 | 3.68E-05 |
| *DR* | TP12 vs TP10 | TP10 | 3.92E-02 |
|  |  |  |  |
| GO:0019538 | BP | protein metabolism | |
| *RN* | TP13 vs TP2 | TP13 | 8.34E-06 |
| *MM* | TP13 vs TP3 | TP13 | 1.99E-05 |
| HS | TP13 vs TP1 | TP1 | 2.08E-02 |
| *DR* | TP12 vs TP10 | TP10 | 1.25E-08 |
| *DR* | TP13 vs TP10 | TP10 | 4.77E-13 |
|  |  |  |  |
| GO:0009790 | BP | embryonic development | |
| *MM* | TP13 vs TP3 | TP13 | 4.96E-02 |
|  |  |  |  |
| GO:0006412 | BP | protein biosynthesis | |
| HS | TP13 vs TP1 | TP1 | 2.78E-04 |
| *DR* | TP12 vs TP10 | TP10 | 4.54E-05 |
| *DR* | TP13 vs TP10 | TP10 | 2.49E-10 |
|  |  |  |  |
| GO:0006629 | BP | lipid metabolism | |
| *RN* | TP13 vs TP2 | TP13 | 2.05E-03 |
| *MM* | TP13 vs TP3 | TP13 | 2.99E-02 |
| *DR* | TP12 vs TP10 | TP10 | 8.98E-03 |
| *DR* | TP13 vs TP10 | TP10 | 5.98E-03 |
|  |  |  |  |
| GO:0003677 | MF | DNA binding | |
| *XT* | TP13 vs TP8 | TP13 | 4.38E-02 |
| *RN* | TP13 vs TP2 | TP13 | 1.20E-08 |
| *MM* | TP13 vs TP3 | TP13 | 1.86E-07 |
| *DR* | TP12 vs TP10 | TP10 | 1.51E-10 |
| *DR* | TP13 vs TP10 | TP10 | 3.99E-10 |
|  |  |  |  |
| GO:0030528 | MF | transcription regulator activity | |
| *DR* | TP12 vs TP10 | TP10 | 4.43E-03 |
| *DR* | TP13 vs TP10 | TP10 | 1.65E-02 |
|  |  |  |  |
| GO:0016049 | BP | cell growth | |
| *MM* | TP13 vs TP3 | TP13 | 4.62E-02 |
| *DR* | TP12 vs TP10 | TP10 | 1.28E-03 |
| *DR* | TP13 vs TP10 | TP10 | 1.80E-02 |
|  |  |  |  |
| GO:0003674 | MF | molecular_function | |
| *DR* | TP13 vs TP10 | TP10 | 2.99E-02 |
|  |  |  |  |
| GO:0000166 | MF | nucleotide binding | |
| *RN* | TP13 vs TP2 | TP13 | 2.26E-11 |
| *MM* | TP13 vs TP3 | TP13 | 3.82E-11 |
| *DR* | TP12 vs TP10 | TP10 | 3.45E-11 |
| *DR* | TP13 vs TP10 | TP10 | 6.45E-15 |
|  |  |  |  |
| GO:0005975 | BP | carbohy*DR*ate metabolism | |
| *RN* | TP13 vs TP2 | TP13 | 3.51E-02 |
| *MM* | TP13 vs TP3 | TP13 | 9.49E-03 |
| HS | TP13 vs TP1 | TP1 | 2.78E-02 |
| *DR* | TP12 vs TP10 | TP10 | 6.66E-05 |
| *DR* | TP13 vs TP10 | TP10 | 1.36E-07 |
|  |  |  |  |
| GO:0006810 | BP | transport | |
| *TN* | TP13 vs TP11 | TP13 | 2.22E-02 |
| *RN* | TP13 vs TP2 | TP13 | 1.51E-11 |
| *MM* | TP13 vs TP3 | TP13 | 1.28E-10 |
| *DR* | TP12 vs TP10 | TP10 | 1.18E-06 |
| *DR* | TP13 vs TP10 | TP10 | 5.92E-07 |
|  |  |  |  |
| GO:0008135 | MF | translation factor activity\, nucleic acid binding | |
| HS | TP13 vs TP1 | TP1 | 1.74E-02 |
|  |  |  |  |
| GO:0004872 | MF | receptor activity | |
| *TN* | TP13 vs TP11 | TP13 | 6.34E-03 |
| *XT* | TP13 vs TP8 | TP13 | 5.83E-03 |
| *RN* | TP13 vs TP2 | TP13 | 2.26E-11 |
| *MM* | TP13 vs TP3 | TP13 | 7.47E-09 |
| *DR* | TP12 vs TP10 | TP10 | 1.01E-08 |
| *DR* | TP13 vs TP10 | TP10 | 7.37E-05 |
|  |  |  |  |
| GO:0030234 | MF | enzyme regulator activity | |
| *TN* | TP13 vs TP11 | TP13 | 2.03E-02 |
| *RN* | TP13 vs TP2 | TP13 | 6.64E-06 |
| *MM* | TP13 vs TP3 | TP13 | 1.33E-03 |
| *DR* | TP12 vs TP10 | TP10 | 4.57E-03 |
| *DR* | TP13 vs TP10 | TP10 | 4.44E-04 |
|  |  |  |  |
| GO:0007267 | BP | cell-cell signaling | |
| *RN* | TP13 vs TP2 | TP13 | 9.15E-05 |
| *MM* | TP13 vs TP3 | TP13 | 5.06E-04 |
| *DR* | TP12 vs TP10 | TP10 | 6.51E-03 |
| *DR* | TP13 vs TP10 | TP10 | 3.03E-03 |
|  |  |  |  |
| GO:0003676 | MF | nucleic acid binding | |
| *RN* | TP13 vs TP2 | TP13 | 6.83E-04 |
| *MM* | TP13 vs TP3 | TP13 | 1.99E-03 |
| HS | TP13 vs TP1 | TP1 | 2.08E-02 |
| *DR* | TP12 vs TP10 | TP10 | 4.69E-10 |
| *DR* | TP13 vs TP10 | TP10 | 1.65E-09 |
|  |  |  |  |
| GO:0003779 | MF | actin binding | |
| *TN* | TP13 vs TP11 | TP13 | 1.97E-02 |
| *RN* | TP13 vs TP2 | TP13 | 2.46E-02 |
| *MM* | TP13 vs TP3 | TP13 | 3.52E-02 |
|  |  |  |  |
| GO:0006464 | BP | protein modification | |
| *RN* | TP13 vs TP2 | TP13 | 8.16E-12 |
| *MM* | TP13 vs TP3 | TP13 | 7.26E-07 |
| HS | TP13 vs TP1 | TP13 | 2.43E-02 |
| *DR* | TP12 vs TP10 | TP10 | 1.00E-09 |
| *DR* | TP13 vs TP10 | TP10 | 2.63E-07 |
|  |  |  |  |
| GO:0005509 | MF | calcium ion binding | |
| *TN* | TP13 vs TP11 | TP13 | 4.93E-02 |
| *RN* | TP13 vs TP2 | TP13 | 9.03E-10 |
| *MM* | TP13 vs TP3 | TP13 | 1.13E-06 |
| *DR* | TP12 vs TP10 | TP10 | 5.37E-04 |
| *DR* | TP13 vs TP10 | TP10 | 1.87E-04 |
|  |  |  |  |
| GO:0005198 | MF | structural molecule activity | |
| *RN* | TP13 vs TP2 | TP13 | 4.11E-02 |
| HS | TP13 vs TP1 | TP1 | 1.65E-03 |
| *DR* | TP12 vs TP10 | TP10 | 9.70E-05 |
| *DR* | TP13 vs TP10 | TP10 | 1.15E-10 |
|  |  |  |  |
| GO:0000003 | BP | reproduction | |
| *RN* | TP13 vs TP2 | TP13 | 4.40E-03 |
| *DR* | TP12 vs TP10 | TP10 | 4.46E-02 |
| *DR* | TP13 vs TP10 | TP10 | 1.90E-02 |
|  |  |  |  |
| GO:0005102 | MF | receptor binding | |
| *RN* | TP13 vs TP2 | TP13 | 1.56E-02 |
| *MM* | TP13 vs TP3 | TP13 | 1.25E-02 |
| *DR* | TP12 vs TP10 | TP10 | 2.33E-05 |
| *DR* | TP13 vs TP10 | TP10 | 4.12E-05 |
|  |  |  |  |
| GO:0006259 | BP | DNA metabolism | |
| *RN* | TP13 vs TP2 | TP13 | 3.51E-02 |
| *MM* | TP13 vs TP3 | TP13 | 1.38E-02 |
| *DR* | TP12 vs TP10 | TP10 | 3.28E-03 |
| *DR* | TP13 vs TP10 | TP10 | 1.11E-06 |
|  |  |  |  |
| GO:0009628 | BP | response to abiotic stimulus | |
| *RN* | TP13 vs TP2 | TP13 | 1.13E-02 |
| *MM* | TP13 vs TP3 | TP13 | 2.53E-03 |
| *DR* | TP12 vs TP10 | TP10 | 4.34E-02 |
| *DR* | TP13 vs TP10 | TP10 | 1.66E-02 |
|  |  |  |  |
| GO:0000004 | BP | biological_process unknown | |
| *RN* | TP13 vs TP2 | TP13 | 1.19E-02 |
| *DR* | TP12 vs TP10 | TP10 | 1.81E-04 |
| *DR* | TP13 vs TP10 | TP10 | 1.15E-03 |
|  |  |  |  |
| GO:0005515 | MF | protein binding | |
| *TN* | TP13 vs TP11 | TP13 | 5.76E-04 |
| *XT* | TP13 vs TP8 | TP13 | 1.01E-02 |
| *RN* | TP13 vs TP2 | TP13 | 2.31E-12 |
| *MM* | TP13 vs TP3 | TP13 | 3.72E-08 |
| *DR* | TP12 vs TP10 | TP10 | 7.78E-07 |
| *DR* | TP13 vs TP10 | TP10 | 4.01E-06 |
|  |  |  |  |
| GO:0016787 | MF | hy*DR*olase activity | |
| *RN* | TP13 vs TP2 | TP13 | 9.22E-07 |
| *MM* | TP13 vs TP3 | TP13 | 1.20E-06 |
| HS | TP13 vs TP1 | TP1 | 1.74E-02 |
| *DR* | TP12 vs TP10 | TP10 | 5.12E-06 |
| *DR* | TP13 vs TP10 | TP10 | 6.19E-13 |
|  |  |  |  |
| GO:0004721 | MF | phosphoprotein phosphatase activity | |
| *RN* | TP13 vs TP2 | TP13 | 6.83E-04 |
| *MM* | TP13 vs TP3 | TP13 | 1.64E-02 |
| *DR* | TP12 vs TP10 | TP10 | 2.26E-03 |
|  |  |  |  |
| GO:0003700 | MF | transcription factor activity | |
| *RN* | TP13 vs TP2 | TP13 | 1.33E-06 |
| *MM* | TP13 vs TP3 | TP13 | 1.38E-07 |
| *DR* | TP12 vs TP10 | TP10 | 6.64E-11 |
| *DR* | TP13 vs TP10 | TP10 | 1.05E-03 |
|  |  |  |  |
| GO:0007582 | BP | physiological process | |
| *RN* | TP13 vs TP2 | TP13 | 2.46E-10 |
| *MM* | TP13 vs TP3 | TP13 | 5.69E-09 |
| *DR* | TP12 vs TP10 | TP10 | 1.78E-04 |
| *DR* | TP13 vs TP10 | TP10 | 5.07E-05 |
|  |  |  |  |
| GO:0009653 | BP | morphogenesis | |
| *RN* | TP13 vs TP2 | TP13 | 4.42E-05 |
| *MM* | TP13 vs TP3 | TP13 | 5.55E-07 |
| *DR* | TP12 vs TP10 | TP10 | 5.96E-06 |
| *DR* | TP13 vs TP10 | TP10 | 4.01E-06 |
|  |  |  |  |
| GO:0030154 | BP | cell differentiation | |
| *RN* | TP13 vs TP2 | TP13 | 3.51E-02 |
|  |  |  |  |
| GO:0016301 | MF | kinase activity | |
| *DR* | TP12 vs TP10 | TP10 | 5.94E-05 |
| *DR* | TP13 vs TP10 | TP10 | 1.22E-05 |
|  |  |  |  |
| GO:0006139 | BP | nucleobase\, nucleoside\, nucleotide and nucleic acid metabolism | |
| *MM* | TP13 vs TP3 | TP13 | 1.58E-02 |
| HS | TP13 vs TP1 | TP1 | 1.41E-02 |
| *DR* | TP12 vs TP10 | TP10 | 2.77E-06 |
| *DR* | TP13 vs TP10 | TP10 | 6.07E-12 |
|  |  |  |  |
| GO:0007275 | BP | development | |
| *TN* | TP13 vs TP11 | TP13 | 3.71E-02 |
| *XT* | TP13 vs TP8 | TP13 | 4.50E-02 |
| *RN* | TP13 vs TP2 | TP13 | 7.64E-08 |
| *MM* | TP13 vs TP3 | TP13 | 1.49E-06 |
| HS | TP13 vs TP1 | TP13 | 1.88E-02 |
| *DR* | TP12 vs TP10 | TP10 | 1.18E-06 |
| *DR* | TP13 vs TP10 | TP10 | 1.06E-03 |
|  |  |  |  |
| GO:0006350 | BP | transcription | |
| *TN* | TP13 vs TP11 | TP13 | 1.97E-02 |
| *RN* | TP13 vs TP2 | TP13 | 7.13E-11 |
| *MM* | TP13 vs TP3 | TP13 | 3.82E-11 |
| *DR* | TP12 vs TP10 | TP10 | 1.54E-20 |
| *DR* | TP13 vs TP10 | TP10 | 4.11E-11 |
|  |  |  |  |
| GO:0007010 | BP | cytoskeleton organization and biogenesis | |
| *RN* | TP13 vs TP2 | TP13 | 7.43E-05 |
| *MM* | TP13 vs TP3 | TP13 | 3.47E-04 |
| *DR* | TP12 vs TP10 | TP10 | 2.66E-02 |
| *DR* | TP13 vs TP10 | TP10 | 6.36E-03 |
|  |  |  |  |
| GO:0016740 | MF | transferase activity | |
| *RN* | TP13 vs TP2 | TP13 | 3.51E-02 |
| *MM* | TP13 vs TP3 | TP13 | 1.31E-03 |
| HS | TP13 vs TP1 | TP1 | 5.96E-03 |
| *DR* | TP12 vs TP10 | TP10 | 1.25E-10 |
| *DR* | TP13 vs TP10 | TP10 | 2.75E-19 |
|  |  |  |  |
| GO:0005215 | MF | transporter activity | |
| *RN* | TP13 vs TP2 | TP13 | 1.63E-06 |
| *MM* | TP13 vs TP3 | TP13 | 9.86E-05 |
| *DR* | TP12 vs TP10 | TP10 | 8.93E-04 |
| *DR* | TP13 vs TP10 | TP10 | 1.65E-08 |
|  |  |  |  |
| GO:0003723 | MF | *RN*A binding | |
| *RN* | TP13 vs TP2 | TP2 | 4.71E-02 |
| HS | TP13 vs TP1 | TP1 | 9.08E-03 |
| *DR* | TP12 vs TP10 | TP10 | 9.36E-04 |
| *DR* | TP13 vs TP10 | TP10 | 3.42E-05 |
|  |  |  |  |
| GO:0006950 | BP | response to stress | |
| *RN* | TP13 vs TP2 | TP13 | 6.83E-04 |
| *MM* | TP13 vs TP3 | TP13 | 1.84E-02 |
| HS | TP13 vs TP1 | TP1 | 2.43E-02 |
| *DR* | TP12 vs TP10 | TP10 | 2.00E-02 |
| *DR* | TP13 vs TP10 | TP10 | 9.92E-07 |
|  |  |  |  |
| GO:0005489 | MF | electron transporter activity | |
| HS | TP13 vs TP1 | TP1 | 8.69E-04 |
| *DR* | TP13 vs TP10 | TP10 | 5.03E-04 |
|  |  |  |  |
| GO:0008283 | BP | cell proliferation | |
| *MM* | TP13 vs TP3 | TP13 | 2.02E-02 |
| *DR* | TP12 vs TP10 | TP10 | 3.34E-04 |
| *DR* | TP13 vs TP10 | TP10 | 1.15E-03 |
|  |  |  |  |
| GO:0040029 | BP | regulation of gene expression\, epigenetic | |
| *DR* | TP13 vs TP10 | TP10 | 2.22E-02 |
|  |  |  |  |
| GO:0008219 | BP | cell death | |
| *MM* | TP13 vs TP3 | TP13 | 2.56E-02 |
| *DR* | TP12 vs TP10 | TP10 | 1.23E-02 |
| *DR* | TP13 vs TP10 | TP10 | 2.03E-04 |
|  |  |  |  |
| GO:0006996 | BP | organelle organization and biogenesis | |
| *RN* | TP13 vs TP2 | TP13 | 4.61E-02 |
| *DR* | TP12 vs TP10 | TP10 | 1.19E-04 |
| *DR* | TP13 vs TP10 | TP10 | 1.57E-08 |
|  |  |  |  |
| GO:0006811 | BP | ion transport | |
| *TN* | TP13 vs TP11 | TP13 | 1.11E-02 |
| *XT* | TP13 vs TP8 | TP13 | 1.79E-02 |
| *RN* | TP13 vs TP2 | TP13 | 1.23E-11 |
| *MM* | TP13 vs TP3 | TP13 | 1.34E-06 |
| HS | TP13 vs TP1 | TP13 | 3.84E-02 |
| *DR* | TP12 vs TP10 | TP10 | 8.52E-03 |
| *DR* | TP13 vs TP10 | TP10 | 1.00E-02 |
|  |  |  |  |
| GO:0030246 | MF | carbohy*DR*ate binding | |
| HS | TP13 vs TP1 | TP1 | 1.56E-02 |
| *DR* | TP13 vs TP10 | TP10 | 3.01E-02 |
|  |  |  |  |
| GO:0005554 | MF | molecular_function unknown | |
| *DR* | TP12 vs TP10 | TP10 | 4.01E-04 |
| *DR* | TP13 vs TP10 | TP10 | 2.33E-04 |
|  |  |  |  |
| GO:0009056 | BP | catabolism | |
| *RN* | TP13 vs TP2 | TP13 | 1.84E-03 |
| *MM* | TP13 vs TP3 | TP13 | 2.24E-04 |
| HS | TP13 vs TP1 | TP1 | 1.74E-02 |
| *DR* | TP12 vs TP10 | TP10 | 8.79E-08 |
| *DR* | TP13 vs TP10 | TP10 | 6.94E-13 |
|  |  |  |  |
| GO:0007049 | BP | cell cycle | |
| *RN* | TP13 vs TP2 | TP13 | 1.79E-04 |
| *MM* | TP13 vs TP3 | TP13 | 8.07E-03 |
| *DR* | TP12 vs TP10 | TP10 | 2.48E-05 |
| *DR* | TP13 vs TP10 | TP10 | 1.42E-06 |
